# May the Force Be with You (Or Not): The Immune System under Microgravity

**DOI:** 10.3390/cells10081941

**Published:** 2021-07-30

**Authors:** Mei ElGindi, Jiranuwat Sapudom, Ibrahim Hamed Ibrahim, Mohamed Al-Sayegh, Weiqiang Chen, Anna Garcia-Sabaté, Jeremy C. M. Teo

**Affiliations:** 1 Laboratory for Immuno Bioengineering Research and Applications, Division of Engineering, New York University Abu Dhabi, Abu Dhabi P.O. Box 129188, United Arab Emirates; me95@nyu.edu (M.E.); jiranuwat.sapudom@nyu.edu (J.S.); ihi2005@nyu.edu (I.H.I.); 2 Biology Division, New York University Abu Dhabi, Abu Dhabi P.O. Box 129188, United Arab Emirates; ma3803@nyu.edu; 3 Department of Mechanical and Aerospace Engineering, New York University, Brooklyn, NY 11201, USA; wchen@nyu.edu; 4 Department of Biomedical Engineering, New York University, Brooklyn, NY 11201, USA

**Keywords:** immune cells, immunology, microgravity, space research, space biology, mechanotransduction

## Abstract

All terrestrial organisms have evolved and adapted to thrive under Earth’s gravitational force. Due to the increase of crewed space flights in recent years, it is vital to understand how the lack of gravitational forces affects organisms. It is known that astronauts who have been exposed to microgravity suffer from an array of pathological conditions including an impaired immune system, which is one of the most negatively affected by microgravity. However, at the cellular level a gap in knowledge exists, limiting our ability to understand immune impairment in space. This review highlights the most significant work done over the past 10 years detailing the effects of microgravity on cellular aspects of the immune system.

## 1. Introduction

Gravitational force plays an important role in developing the functions and characteristics of all terrestrial organisms. Understanding the effects of gravity on organisms is vital if human space exploration and future colonization are to expand beyond low Earth orbit, which will greatly increase exposure time to microgravity conditions (×10^−6^ g), as well as other gravity levels such as the Moon’s (0.16 g) or Mars’ (0.37 g) gravity. It has been known for decades that lack of these gravitational forces has detrimental physiological effects on the human body. Astronauts who are exposed to microgravity for prolonged periods of time have been found to suffer from decreased neurological function, bone density decline, atrophied muscles, and significantly compromised immune systems upon their return to Earth [1,2,3,4,5,6,7]. In terms of bone health, studies showed that astronauts could lose up to 1.5% of their bone mass every month they spend in space [2,8,9,10,11,12,13,14]. Cancer is also a major risk for astronauts due to the changes in gene expression caused by concomitant exposure to radiation as well as the varying gravitational forces [15,16,17]. Research on the brain shows that astronauts are at greater risk of developing neurological problems such as increased intracranial pressure, visual impairments, and spaceflight-associated neuro-ocular syndrome [15,18,19,20].

One of the greatest physiological impacts that long term exposure to microgravity has is on the immune system. It is known that immune cell function, morphology, and differentiation are impaired in the absence of gravity [5,6,21,22,23,24,25]. Studies dating back nearly fifty years showed that up to 50% of astronauts returning from space since the Apollo missions have a compromised immune system and are prone to bacterial and viral infections shortly after returning to earth [26]. As the duration of crewed space missions extends, it is becoming increasingly important to understand the role that gravity plays on the immune system, to ensure the well-being of astronauts for the success of these missions. Findings in this field will not only benefit astronauts, but they can also provide a better understanding on the deterioration of the immune system due to aging back on Earth since age-related processes have been reported to be accelerated in microgravity [27,28].

This review will provide a brief overview of the existing microgravity platforms and then highlight the most prominent studies in the past decade to understand the effects of gravity on the immune system. These studies are important to help understand the impact of gravitational forces on the cellular functions and developmental mechanics of immune cells. This review will also provide insight into the aspects of the immune system in microgravity that have yet to be addressed.

## 2. Real and Simulated Microgravity Platforms

**Figure 1:**
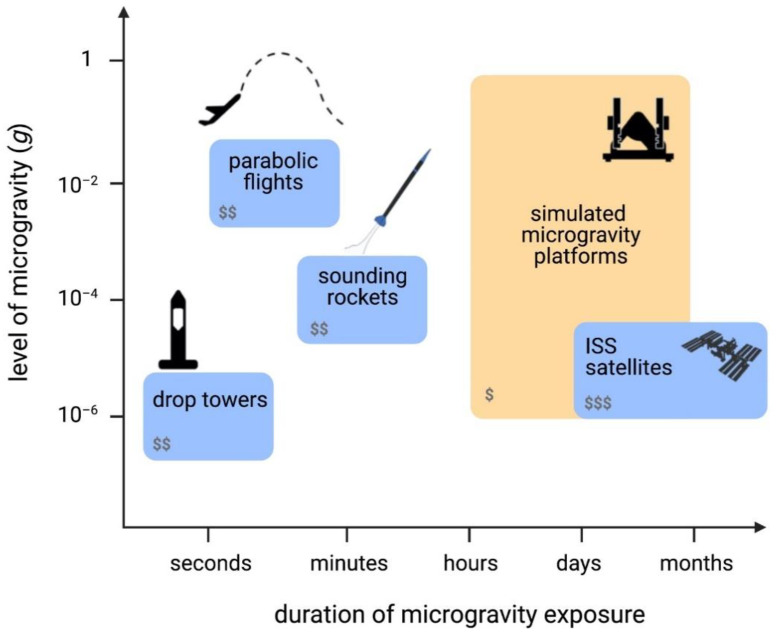
An overview of microgravity platforms. Overview of real (blue) and simulated (yellow) microgravity platforms in terms of duration of microgravity exposure and level of microgravity reached compared to Earth g (where g = 9.81 m/s^2^). Number of $ represents relative cost of running experiments on each platform.

Several platforms to obtain microgravity are available, each having different magnitudes and time duration of applied microgravity, and complexity of experimental implementation. We will distinguish between platforms to obtain real microgravity, whether it is on ground or in orbit, and simulated microgravity, which is obtained by leveraging the delay of biological organisms in sensing gravity (Figure 1). When choosing a microgravity platform for any experiment, the time-scale of the experiment is a critical aspect to take into consideration, and biological processes can generally span from a few minutes to several weeks [29].

Real microgravity platforms include parabolic flights, which alternate between microgravity and hypergravity (2 g) during each parabola [30]. This platform can provide about 20 s of microgravity conditions per parabola with up to about 30 parabolas per flight, and a microgravity level of 10^−2^ g (where g = 9.81 m/s^2^) [30]. While parabolic flights are a suitable platform for certain aspects of human physiology research due to their large volume, the short intervals of microgravity make them less than ideal for biological experiments that require longer periods of microgravity exposure for measurable results.

A second platform to study real microgravity is the drop tower. Drop towers can provide very good quality of microgravity (10^−6^ g) at the expense of experimentation time, which is extremely short, ranging from 2.2 to 9.3 s. Sounding rockets, also referred to as suborbital ballistic rockets, can offer 5 to 15 min of microgravity at levels no higher than 10^−4^ g [30]. However, they commonly have very limited payload space available, making experimental design very complex, and duration is unsuitable for longer in vitro or in vivo experiments. Moreover, the high levels of hypergravity during launch and re-entry, as well as high spin rates of suborbital rockets, can have negative impacts on the samples [31].

Finally, orbital platforms, from satellites to the International Space Station (ISS), can also provide real microgravity and they can offer long duration at microgravity levels of 10^−6^ g [30]. Although it is possible for astronauts to have (limited) interaction for experiments on the ISS, it is strongly desirable in both platforms to have a high degree of automation, which will increase the complexity of experimental setups. Access to these platforms is usually costly, and they require very long experimental planning, an iterative development cycle, and lengthy integration times. In recent years, access to space using CubeSats (small satellites) has become more affordable, making CubeSats a potentially suitable platform for biology experiments. However, their size is a limiting factor, posing technical challenges demanding the miniaturizing of common processes required for benchtop biology experiments (e.g., media exchanges, freezing, etc.) and equipment (e.g., brightfield and fluorescence microscopes, flow cytometers, etc.), whilst needing full automation of experiments.

Simulated microgravity can be achieved through a number of platforms that rely on the delay of organisms to sense gravity, as well as their time of sedimentation. By means of rotation under different conditions, they average the vector of gravity to zero over a cycle, making the samples perceive an environment of microgravity. In all cases, the samples must be as close to the center of rotation as possible to prevent any undesired fluid dynamics due to centrifugal forces [32], which need to be taken into consideration during data analysis. These platforms can provide valuable data and are attractive to use as they allow extended experimental time at low cost. Ultimately, they are not a substitute for real microgravity, and results should be further validated in any of the real microgravity platforms [33]. Simulated microgravity is extremely attractive for optimizing microgravity-focused experimental conditions as a first pass prior to real microgravity. The systems in this category include 2D and 3D clinostats, the Rotating Wall Vessel (RWV), also known as the Rotary Cell Culture System (RCCS), and the Random Positioning Machine (RPM) (Figure 1). Clinostats rotate at constant speed either around one axis (2D) or on two axes (3D). RWVs use faster rotation speeds to compensate for sample sedimentation, and thus they are more suitable for suspension cells. However, adherent cells have also been studied before on this platform by culturing them on beads [34]. The RPM rotates on two axes, similarly to the 3D clinostat. However, in this case the two independent frames rotate at different speeds and directions, whereas the 3D clinostat moves at a constant speed in a constant direction.

## 3. Immune Cells and Microgravity

**Figure 2:**
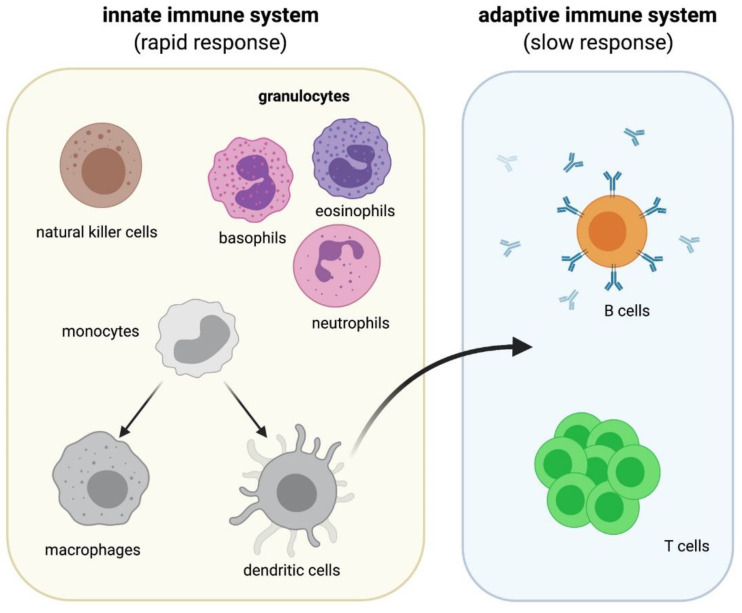
Overview of immune cells in the innate and adaptive immune systems. The schematic provides an overview of the two arms of the immune system: the innate immune system and the adaptive immune system. The innate immune system consists of granulocytes (basophils, neutrophils, and eosinophils), natural killer cells, monocytes, macrophages, and dendritic cells. The adaptive immune system consists of T cells and B cells with dendritic cells being the main bridge between the two systems.

The immune system is an expansive and complex network of cells that protects our body against infection. Here, we provide a brief overview of the immune system as detailed reviews of the immune system are already in published work [35,36,37,38] and constantly updated. The human immune system is divided into two parts: the innate immune system and the adaptive immune system (Figure 2). These two systems are highly interconnected and will not serve their complete functions to safeguard our bodies without one another. Microgravity is known to have effects on both parts of the immune system [6,15,39]. In this section, we will discuss the effects of microgravity on the innate immune cells that are involved in phagocytosis of invading pathogens, antigen presentation, and cytokine production as well as the cells of the adaptive immune system.

### 3.1. Microgravity Studies on Cells of the Innate Immune System

The innate immune system is the first line of defense against pathogens. It is made up of granulocytes, natural killer (NK) cells, monocytes, macrophages, and dendritic cells (DCs) (Figure 2). Neutrophils, basophils, and eosinophils are all considered granulocytes; neutrophils are the most abundant granulocyte in the immune system. They are an essential part of the innate immune system and work primarily by phagocytizing pathogens in the blood and tissue. NK cells are a critical subset of the innate immune system and are involved in killing virus infected cells and also react to curb tumor formation. During inflammation, blood borne monocytes are recruited to sites of inflammation and can differentiate into DCs and macrophages. The dysregulation of either cell type can result in chronic infections, blood disorders, autoimmune disorders, and certain cancers, indicating the important role that they play in the immune system [40,41]. DCs are also essential in priming the adaptive immune response which is the second line of defense against infection (Figure 2). Along with the detailed review of each cell type below, briefly summarized observations of the effects of microgravity on the innate immune cells can be found in Table 1.

#### 3.1.1. Neutrophils

To date, very little work has been done with regard to the effects of microgravity on neutrophil functions both in vivo and in vitro. The several studies on neutrophils have focused on neutrophil numbers present in blood, with a reported increase in circulating neutrophils in humans returning from spaceflight [42,43]. In line with this, a more recent study using 30 parabolas of parabolic flights, with a duration of 20 s of microgravity in each, showed that neutrophil levels in human blood are slightly increased, whereas all other immune cell subsets, such as T cells and B cells, decreased [44]. Similarly, an increase in neutrophils and neutrophil to lymphocyte ratio (NLR) has been reported in humans during a 180 day spaceflight as well as in human PBMCs cultured for 20 h on an RWV, at 20 revolutions per minute (rpm), indicating that NLR could be used as a potential biomarker to monitor in flight health [45]. Alternatively, a spaceflight (up to 15 days) study found no change in overall neutrophil levels in sampled human blood before and after flight [46]. Although most studies point towards increased neutrophil levels under microgravity, discrepancies in results could be due to specific characteristics of spaceflight (such as duration) or variability of donors.

#### 3.1.2. Natural Killer (NK) Cells

The effects of simulated microgravity on primary human NK cells have been studied by means of RWV. The research project investigated several characteristic attributes such as receptor expression and cytokine secretion. The authors found that there was a decrease in NK cell cytotoxicity after 48 h under simulated microgravity conditions, at 30 rpm, that was related to a decrease in associated cytokine production and surface receptor expression [47]. Specifically, NKG2D, an activating receptor found on the surface of NK cells, was found to be significantly reduced. In 2019, a study investigated NK cell function isolated from astronaut peripheral blood that underwent a 6 month mission on the ISS [48]. The results showed impaired NK cell function in terms of cytotoxic activity against leukemia K562 cell line and this impairment was more prominent in astronauts who underwent their first flight compare to experienced astronauts, possibly indicating possible epigenetic adaptation [48]. Another study found that polysaccharides, which are known stimulants of NK cells, were able to restore the expression of NKG2D under RWV simulated microgravity conditions at 30 rpm [49]. These studies suggest that polysaccharides could be useful in promoting a more functional immune response in space.

#### 3.1.3. Monocytes

Few studies have addressed the effect of microgravity on monocytes. A study examined the peripheral blood of nine astronauts and found that the total monocyte percentages were unchanged after a 13–16 day spaceflight [50]. However, expression of CD62L and HLA-DR was reduced indicating an impairment in monocyte adhesion to vasculature, tissue migration, and antigen presentation. Following lipopolysaccharide (LPS) stimulation, secreted pro-inflammatory cytokines, namely IL-6, TNFα, and IL-10 were reduced, indicative of further impaired monocyte function and inflammatory response during spaceflight [50]. Using parabolic flight and sounding rocket real microgravity conditions, U937 monocytic cells were used to find potential gene targets for standardizing protocols for microgravity studies. Microarray studies identified eight genes (*ALB*, *B4GALT6*, *GAPDH*, *HMBS*, *YWHAZ*, *ABCA5*, *ABCA9*, and *ABCC1*) that remained stable during normal and microgravity conditions [51]. This study is significant in providing a step forward in optimizing protocols for microgravity studies. Albeit reserved for U937 cells, discrepancies in results can be avoided by assessing these genes when employing different microgravity platforms and cell handling.

#### 3.1.4. Macrophages

Macrophages are differentiated from monocytes and can be activated into different subtypes. The main subtypes are pro-inflammatory (M1) and anti-inflammatory (M2) macrophages. In many in vitro investigations, monocytic cell lines (e.g., THP-1, U937, J-111) are differentiated into uncommitted macrophages using Phorbol 12-myristate 13-acetate (PMA) and activated into specific subtypes using interferon-gamma (IFNγ) and LPS for M1, or IL-4 and IL-13 for M2 [52,53]. Notably, PMA also activates other immune cells (e.g., T cells) in a non-specific manner.

Studies have shown that non stimulated U937 cells respond to both parabolic flight and 2D clinostat simulated microgravity at 60 rpm by having increased tyrosine phosphorylation and activated c-jun, whereas PMA stimulated U937 were seen to have the opposite effect, with reduced tyrosine phosphorylation and activation of c-jun [26]. These results indicate that microgravity conditions cause non-specific activation of monocytic U937 cells and that further immune activation via PMA is inhibited under these conditions. Other studies also revealed that PMA stimulated U937 cells had a disorganized cytoskeleton with a significant decrease in actin expression, a cytoskeletal protein, under microgravity conditions. The cells also showed a decreased expression of CD18, CD36, and MHC-II, proteins involved in adhesion, scavenging, and antigen presentation, respectively, after 5 days of spaceflight [54]. These data imply an overall decreased function of macrophages under microgravity conditions. Similarly, under real spaceflight conditions of microgravity, J-111 cells were found to have decreased cell motility and a reduction in fluorescence intensity of F-actin in the cells [55]. The cytoskeleton and surface proteins are critical for macrophage morphology, motility towards pathogens, and function and these data indicate that spaceflight and microgravity impair host immune defense.

A study by Paulsen et al. showed that under real microgravity (30 parabolas per parabolic flight and 5 days in spaceflight) and 2D clinostat simulated microgravity (up to 5 days at 60 rpm) conditions, both PMA differentiated U937 human macrophages and human primary M2 macrophages had an increased expression of intracellular adhesion molecule 1 (ICAM-1) [56]. This study also indicated that murine BV-2 microglial cells had a decrease in ICAM-1 expression under simulated microgravity and undifferentiated U937 cells showed no changes in ICAM-1 levels under any microgravity conditions [56]. The differences observed could be due to species variation or peripheral vs. central nervous system macrophages; however, ICAM-1 could be considered a marker for rapid-response to changes in gravity. Conversely, a study by Tauber et al. reported that human primary M1 macrophages showed a decrease in ICAM-1 expression after 11 days in real microgravity conditions on board the ISS [57]. No structural changes to actin or vimentin were seen during this time and an increase in free fucose was also shown along with a decrease in cell surface bound fucose. These effects could contribute to functional impairment of macrophages and inability to efficiently activate T cells. The authors also conclude that the lack of significant changes in cell cytoskeleton could reveal a steady state adaptive process to microgravity [57]. Another study showed that human primary M1 macrophages under real microgravity conditions of suborbital ballistic rockets were found to have a significant reduction in cell volume, nuclear volume, and actin cytoskeleton as early as 4 s in microgravity as opposed to the 11 days seen in the earlier experiment [58]. This showed that there is a rapid response of macrophages to microgravity conditions and that cytoskeleton rearrangement or dysregulation is one of the primary effects of microgravity.

Primary mouse macrophages that were induced with LPS were found to have significantly decreased TNFα levels, but not IL-1β levels after short term culture (24 h) in an RCCS simulated microgravity system [59]. The molecular studies showed that the intracellular signaling pathway of LPS was not affected by microgravity and that heat-shock factor 1 (HSF1), a repressor of the TNFα promoter, was highly activated under these conditions [59]. The results suggest that microgravity affects the signaling pathways of TNFα and IL-1b via different mechanisms. The same RCCS simulated microgravity system, used at 12–25 rpm, was determined to induce the overexpression of C/EPBb, an activator of arginase, in primary mouse macrophage cells [60]. These cells also had increased levels of p38 MAPK, which could lead to the increase in C/EBPb [60]. When these cells were stimulated with LPS, IL-6 levels were increased in simulated microgravity, compared to controls, and IL-12b was down-regulated [60]. Increased arginase levels could have immunosuppressive effects on macrophages and less IL-12b results in dysregulated differentiation of T cells. IL-6 plays several roles in macrophage function, but increased levels could result in impaired neutrophil recruitment to the site of infection. Together these data indicate that microgravity affects macrophage function on a molecular level by disrupting various signaling pathways.

Using a 2D clinostat simulated microgravity system at 60 rpm, Brungs et al., have reported that the production of ROS by LPS stimulated NR8383 rat macrophages is diminished under microgravity conditions after 50 min [61]. This was caused by decreased SYK phosphorylation, which is required for ROS production. If this dysregulation is also present in real microgravity for humans, it could explain astronauts increased susceptibility to infections [62]. Thiel et al. were able to demonstrate the first direct evidence of rapid cellular sensitivity to gravity using NR8383 rat macrophages on the ISS [63]. They found that these cells are able to adapt to microgravity conditions within approximately 30 s. ROS oxidative burst levels were found to decrease immediately once microgravity conditions were achieved but quickly re-adapted back when returned to 1 g conditions [63]. This indicates the potential for macrophages to rapidly adapt to varying gravity conditions. In addition, the NADPH oxidase complex is involved in ROS levels in the cells as well as with the cytoskeleton [64]. This links the microgravity effects on the macrophages back to cytoskeletal disruption discussed earlier in the section.

More recently, gene expression data that showed both real microgravity on 12 day spaceflights and RCCS simulated microgravity (24 rpm) caused a reduction in primary mouse macrophage differentiation and functionality [65]. The data revealed that microgravity decreased the differentiation of hematopoietic progenitor cells into M1 and M2 macrophages [65]. The authors also implicated the RAS/ERK/NFκB pathways as potential targets to combat the detrimental effects microgravity has on M1 macrophages due to the fact that exogenous ERK and NFκB activators were able to counteract the effects of gravity on the macrophages [65]. Wang et al., used RNA-Seq and similarly found that RCCS simulated microgravity (72 h at 18 rpm) significantly suppresses the production of inflammatory cytokines such as IL-6 and TNFα as well as the MAPK/ERK pathways in stimulated U937 macrophage cells [66]. Microgravity plays an important role in how macrophages respond to infections as seen by the studies above. Real microgravity and simulated microgravity studies show that it affects multiple pathways and processes within the cells that impairs their ability to provide an adequate immune response.

#### 3.1.5. Dendritic Cells (DCs)

Besides macrophages, monocytes also differentiate into dendritic cells during inflammation. Similar to macrophages, monocytic cells lines (e.g., THP-1, U937, J-111) can be differentiated into dendritic cells using IL-4 and granulocyte-macrophage colony-stimulating factor (GM-CSF) [67,68,69]. They can be further matured using various antigens, including LPS, TNFα as well as viral particles [70,71]. Unlike macrophages, few studies on DCs response to microgravity have been conducted, which is surprising since DCs are the main mediators of the adaptive immune response.

Under simulated microgravity conditions on an RCCS at 16 rpm, it was seen that short term culture (<72 h) of JAWS II DCs (a murine DC cell line) resulted in increased expression of surface markers such as (pSTAT-5, mTOR, GM-CSF, MHCII, CD80 (unstimulated)) and IL-6 production [72]. These changes increased the DCs capability to activate both CD4 and CD8 T cells measured by IL-2 and IFNγ production. Prolonged culture (4–14 days) in simulated microgravity, however, reduced these expression markers and the ability to activate T cells [72]. This provides further insights into how the innate immune system can be compromised by long term spaceflight. RCCS simulated microgravity was also used to determine the effects of development of DCs from human hematopoietic stem cells (HSCs) under microgravity conditions [73]. Low et al. found that plasmacytoid DCs (pDCs) and myeloid DCs (mDCs) (two subtypes of DCs) had greater numbers under normal gravity conditions compared to microgravity after 21 days in culture [73]. These results indicate that spaceflight could impair the development of DCs and greatly suppresses the ability for humans exposed to microgravity to launch an immune response.

**Table 1:** Overview of the effects of microgravity on cells of the innate immune system.

Cell Type	Gravity Type	Platform	Cell Source	Observation	Ref.
Neutrophil	Real	Parabolic Flight	Human PBMC	Neutrophils are increased while all other immune cell subsets decrease	[44]
		Spaceflight	Human PBMC	No change in neutrophil numbers before and after flight	[46]
	Real/ Simulated	Spaceflight/ RWV	Human PBMC	Increase in neutrophils and neutrophil to lymphocyte ratio which could be used as a potential biomarker to monitor in flight health	[45]
NK	Simulated	RWV	Human PBMC	Decrease in NK cell cytotoxicity related to a decrease in associated cytokine production and surface receptor expression	[47]
		RWV	Human PBMC	NKG2D was found to be significantly reduced on the surface of NK cells and rescued with the addition of polysaccharides	[49]
	Real	ISS	Human PBMC	Impaired NK cell function in terms of cytotoxic activity against leukemia K562 cell line	[48]
Monocytes	Real	Spaceflight	Human PBMC	Total monocyte percentages were unchanged after a 13–16-day spaceflight	[50]
		Parabolic Flight and Sounding Rocket	U937	Microarray studies identified eight genes that remained stable during normal and microgravity conditions	[51]
Macrophages	Real	Spaceflight	J-111	J-111 cells were found to have decreased cell motility and a reduction of F-actin	[55]
		Spaceflight	U937	PMA stimulated U937 cells had a disorganized cytoskeleton and decreased expression of CD18, CD36, and MHC-II	[54]
		ISS	Human Primary M1	M1 macrophages showed a decrease in ICAM-1 expression. No structural changes to actin or vimentin were seen and an increase in free fucose was also shown along with a decrease in cell surface bound fucose	[57]
		Sounding Rocket	Human Primary M1	Human primary M1 macrophages were found to have a significant reduction in cell volume, nuclear volume, and actin cytoskeleton as early as 4 s in microgravity	[56]
	Real/ Simulated	Parabolic Flight/ 2D Clinostat	U937	Microgravity conditions cause non-specific activation of U937 cells and PMA stimulated U937 cells are inhibited under these conditions	[26]
		Parabolic Flight/Spaceflight/2D Clinostat	U937/ Human Primary M2	U937 human macrophages and human primary M2 macrophages had an increased expression of ICAM-1	[54]
DCs	Simulated	RCCS	Human PBMC	pDC and mDC number are decreased under microgravity indicating that spaceflight could impair the development of these cells	[73]
		RCCS	JAWS II DC	Prolonged culture of DCs in simulated microgravity reduced surface expression markers and the ability to activate T cells	[72]

### 3.2. Microgravity Studies on Cells of the Adaptive Immune System

The adaptive immune system consists of lymphocytes: T cells and B cells (Figure 2). T cells are essential cells that can develop into several subsets with each having different functions from killing unhealthy host cells to secreting cytokines that provide signals to B cells. The two main subsets of T cells are CD4+ T cells and CD8+ T cells. After contact with DCs, CD4+ T cells can proliferate and differentiate into different effector subsets of T helper cells (Th1, Th2, Th17, Treg) depending on the cytokines found in their immediate microenvironment, predominantly secreted by the antigen presenting cells (APCs) they are in contact with. Different subtypes of CD4+ T cells are characterized by their cytokine secretion profile and are distinctively involved in protection against infection and further regulation of T cells. CD8+ T cells recognize peptides presented on DCs and destroy undesirable host cells by secretion of Fas ligand (FasL), cytotoxic granules (perforin and granzymes), and cytokines (e.g., TNFα and IFNγ) [74]. Disruption of T cell development and function is one of the primary factors that can lead to autoimmune diseases such as rheumatoid arthritis and type 1 diabetes [75,76]. B cells are responsible for producing antigen specific antibodies to help fight off infections. Dysregulation of B cells leads to a wide variety of autoimmune diseases such as multiple sclerosis and lupus erythematosus [77,78,79]. Along with the detailed review of each cell type below, briefly summarized observations of the effects of microgravity on the adaptive immune cells can be found in Table 2.

#### 3.2.1. T Cells

Relative to other immune cells, the effects of microgravity on T cell response have been more extensively studied. In vitro experiments to investigate T cell response use primary cells and cell lines (e.g., human Jurkat T cells and transgenic OT-II mouse cells). Several studies have shown that T cells repeatedly fail to proliferate or secrete cytokines in response to T Cell Receptor (TCR) agonists, such as concanavalin A (conA) and anti-CD3 antibody, during spaceflight [80,81,82]. This lack of response was found to be rescued when mitogenic stimulation with PMA was added and was thought to indicate an important role for diacylglycerol (DAG) signaling in T cell response to microgravity [82,83,84]. In a follow up publication, Simons et al. then concluded that there was no support for this claim and found that there was no impairment of DAG, or further downstream signaling, in CD4+ PBMCs by culture in simulated microgravity using the RWV at 14 rpm [85]. These studies highlight the discrepancies in results when using different microgravity platforms. A 2012 study by Thiel et al. showed that anti-CD3 stimulated, and co-stimulated with anti-CD28 as seen physiologically, human primary and Jurkat T cells under both 2D clinostat simulated microgravity at 60 rpm and parabolic flight real microgravity conditions had disrupted cell cycle regulatory proteins such as p21^Waf1/Cip1^, cdc2, and cdc25C [86]. The mRNA expression of cell cycle arrest protein p21 increased 4-fold within 20 s in anti-CD3/anti-CD28 stimulated primary T cells and 2.9-fold in Jurkat T cells under real microgravity. This phenomenon was able to be reverted by the addition of curcumin, which is a histone acetyltransferase inhibitor. This data indicated that microgravity has a negative effect on the cell cycle process in human T cells. Additionally, it was found that conA and anti-CD28 activated T cells from human PBMCs, in both real microgravity (ISS) and simulated microgravity on the RWV rotating at 14 rpm, had significantly downregulated gene expression of Rel/NF-B transcription factors [87]. This leads to a decrease in downstream effectors involved in T cell activation. In addition, CD83 and CD69, early activation signs in T cells, were found to have almost twofold lower expression in microgravity compared to controls which furthers the point that microgravity has an effect on lymphocyte proliferation [87]. CREB1 and SRF-binding sites also had lower expression in T cells under microgravity again implying its effects on the T cell activation process [87]. In both non-stimulated and PMA and anti-CD3/anti-CD28 stimulated Jurkat T cells under 2D clinostat rotation at 60 rpm or parabolic flight conditions, there was an increase in the phosphorylation of MAP kinases ERK-1/2, MEK, and p38 which are involved in the signal transduction cascade in T cells [26]. Increased phosphorylation of MAP kinases could lead to a decrease in T cell response upon TCR engagement [26]. In accordance with these data, Tauber et al. found reduced expression of CD3, IL-2 receptor (IL-2R), and p44/42-MAPK-phosphorylation, all indicators of T cell activation, in primary human conA and anti-CD28 activated T cells undergoing a 6 min microgravity flight [88]. They showed similar results in conA and anti-CD8 activated human T cells using both real microgravity on parabolic flights and simulated microgravity, in 2D clinostat at 60 rpm, along with decreased Zap-70 expression, which is critical for T-cell signaling [89]. These data emphasize that T cell signaling pathways and their functions are negatively affected during exposure to different microgravity platforms.

In addition to microgravity playing a role in T cells function, time of exposure to microgravity has also been implicated in how T cells are affected. Luo et al. have shown that T cells isolated from mouse spleens that underwent differential time exposure to rotary bioreactor simulated microgravity, at 10 rpm, have different responses to conA stimulation [90]. T-cell activation markers such as CD25, CD69, and inflammatory cytokines (IL-2 and IFNγ) were all decreased in a time dependent manner from 24 to 72 h with conA stimulation under simulated microgravity [90]. It was also seen that CD4+ T cells were more susceptible to simulated microgravity effects of decreased proliferation than CD8+ T cells [90]. Decreased proliferation of CD4+ T cells results in less functional subsets and cytokine production and will weaken immune response against infections. Due to the different roles that CD4+ and CD8+ T cells play in the immune system. This data provides further insight into functional differences created by microgravity exposure on the cell subtypes.

Gene expression studies are important for understanding the network and correlation between genes that are affected by changes in environment. One of the first studies that showed that miRNA expression was altered during spaceflight on the ISS indicated that gene expression of miR-21, which is involved in cell cycle progression and proliferation, was suppressed in conA and anti-CD28 stimulated primary human T cells [91,92]. Microarray analysis also showed that 85 genes, several of which are targets of and regulated by miR-21, were significantly suppressed in these cells [92]. Showing that miRNA is also altered during spaceflight suggests that gravity does not only have an effect on T cell activation by suppressing transcription factors, but also by blocking noncoding RNA. Thiel et al. used RNA-Seq and found that a greater number of genes were upregulated than down-regulated in non-activated Jurkat T cells [93]. Additionally, genes were upregulated after only 20 s of microgravity under parabolic spaceflight, indicating that human cells are highly dynamic in their reaction to new gravitational environments [93]. On the other hand, they also performed a 5 min suborbital ballistic rocket experiment which revealed more genes that were downregulated than upregulated. There were more differentially expressed genes in the 5 min suborbital ballistic rocket microgravity exposure than in the 20 s parabolic flight exposure [93]. These differences could be due to the duration of exposure to microgravity, or the gravity level reached by the different platforms (Figure 1). The gene changes were found primarily in regulatory RNA showing that these play an initial role in adaptation to gravity levels [93]. This study also pointed at the importance of having optimized and standardized procedures for studying microgravity effects on cells given the different results that are obtained using different platforms. A similar study showed that there were five genes (*ABCA5*, *GAPDH*, *HPRT1*, *PLA2G4A*, and *RPL13A*) that remained unaltered in microgravity conditions during a 20 s parabolic flight, 5 min suborbital ballistic rocket flight, and 5 min 2D clinostat simulated microgravity at 60 rpm exposure in non-activated Jurkat T cells [94]. These genes will provide good reference genes for future studies and the authors also suggest that microgravity does not affect gene expression homeostasis more than other environmental stressors such as heat shock, exercise, and exposure to radiation [94]. Recently, it was shown that 11 transcript clusters (TCs) in non-activated Jurkat T cells were altered during 5 min under real microgravity conditions on a suborbital ballistic rocket and after 5 min under simulated microgravity on a 2D clinostat rotating at 60 rpm [95]. These common altered TCs were only 1% of the total TCs found to be changed after suborbital ballistic rocket flight indicating that there should be a more standardized method to study mechanical forces in cell culture [95]. Another gene study showed that primary mouse T cells activated with anti-CD3 and anti-CD28 in spaceflight for 15 days, or under simulated microgravity on an RWV and RPM for 2.5 h, had suppressed immune regulatory genes compared to controls [96]. All three platforms were compared in relation to qPCR expression of six genes (*Il2*, *Il2r*, *Ifn*, *Iigp1*, *Slamf1*, and *Tagap*) that are expressed early on in T cell activation and they were found to be suppressed under all microgravity platforms [96]. It is important to note that these gene expression studies require further investigation into the functional effects to more specifically determine the role microgravity is playing on the cells.

In 2015, a study used OT-II mice, which have transgenic CD4+ T cells specific for the OVA peptide, to show that T cell tolerance in vivo was suppressed during a 15 day spaceflight [97]. Flight mice showed a 2-fold increase in OT-II cells compared to control as well as a significant increase in proinflammatory cytokines, such as IL-1b and IL-17, release when harvested cells were restimulated with OVA in vitro [98]. Similarly, one of the first studies to look at T cell and DC interaction in simulated microgravity found that long term culture (5 days) of OT-II T cells in RCCS at 14 rpm results in their resistance to activation by JAWS II DCs [99]. The authors found an increase in CTLA-4, which controls T cell proliferation, levels on the surface of OT-II T cells, and when blocked, activation was restored indicating that CTLA-4 expression may contribute to this phenomenon [99]. In a more recent study, Bradley et al. showed that murine lymphoma cells were able to produce factors that prevent DCs from activating CD4+ T cells [100]. Under simulated microgravity on an RCCS at 16 rpm for 72 h, the IL-2 production by CD4+ T cells was slightly restored and CD8+ T cell responsiveness was increased compared to controls showing that simulated microgravity could help prevent tumor mediated escape and make the cancer cells more susceptible to T cells [100]. More studies are needed to further understand the effects of microgravity on T cell interactions with DCs and other cells of the immune system since this is a critical in vivo step to mount an adequate immune response.

Non-activated Jurkat cells placed under simulated microgravity conditions in an RPM at 60 deg/s were found to have decreased Ca^2+^ and ROS levels compared to 1 g controls up to 24 h under these conditions [101]. The results further suggest that by 96 h, these cells adapt to the new environment and return to control levels [101]. In another metabolic study, authors found that although HIF1α is significantly reduced in non-activated Jurkat cells during the hypergravity phase of parabolic flights, the levels of HIF1α remained relatively unchanged during microgravity exposure [102]. This could potentially be due to the short exposure time to microgravity of parabolic flights and the effects of long-term exposure should be further investigated to better understand the metabolic changes of the cells under microgravity.

#### 3.2.2. B Cells

A 15 day real spaceflight study showed no changes in B cell levels in the peripheral blood of astronauts [46]. In accordance with this, a 6 month spaceflight study showed no change in overall B cell subset numbers or proportions or in levels of plasma immunoglobulins (Igs) [103]. However, Tascher et al. found a significant reduction in B cells in the spleens of mice 1 week after landing from a 1 month flight in space [104]. These differences could be species related. This decrease of B cells was also seen in a parabolic flight real microgravity setting in human peripheral blood [44]. Microgravity and radiation are two of the main factors that influence astronauts’ health while in space. Because these effects are difficult to recreate simultaneously, it is difficult to study them in vivo in real microgravity or in vitro in simulated microgravity. Only one study has demonstrated the effect of microgravity and radiation on B cells. Dang et al. found that simulated microgravity on an RWV decreased ion-radiation generated cell survival and increased apoptosis in human B lymphoblast HMy2.CIR cells [105]. These conditions also increased radiation induced intracellular ROS generation [105]. However, less is known about the extent to which microgravity affects antigen-specific response and antibody production of B cells.

**Table 2:** Overview of the effects of microgravity on cells of the adaptive immune system.

Cell Type	Gravity Type	Platform	Cell Source	Observation	Ref.
T cells	Simulated	RWV	Human PBMC	Microgravity causes no impairment of DAG, or further downstream signaling, in CD4+ T cells	[85]
		RCCS	Mouse Primary	CD25, CD69, IL-2 and IFNγ were all decreased in a time dependent manner from 24 to 72 h under simulated microgravity. CD4+ T cells were more susceptible to simulated microgravity effects of decreased proliferation than CD8+ T cells	[90]
		RCCS	OT II mice	Long term culture of OT-II T cells results in resistance to activation by JAWS II DCs	[99]
		RPM	Jurkat T cells	Decreased Ca^2+^ and ROS levels compared to 1 g controls	[101]
	Real	Spaceflight	Human PBMC	Reduced expression of CD3, IL-2R and p44/42-MAPK-phosphorylation	[88]
		ISS	Human PBMC	Gene expression of miR-21 was suppressed in conA and anti-CD28 stimulated T cells	[92]
		Spaceflight	OT II mice	2-fold increase in OT-II cells in microgravity and an increase in IL-1b and IL-17 release when the cells were restimulated with OVA in vitro	[98]
		Sounding Rocket/ Parabolic Flight	Jurkat T cells	Gene changes were found primarily in regulatory RNA	[93]
		Parabolic Flight	Jurkat T cells	Levels of HIF1a remained relatively unchanged during microgravity exposure	[102]
	Real/ Simulated	2D Clinostat/ Parabolic Flight	Jurkat T cells	Increase in the phosphorylation of MAP kinases ERK-1/2, MEK, and p38	[26]
		2D Clinostat/ Parabolic Flight	Human PBMC/Jurkat T cells	Microgravity disrupted cell cycle regulatory proteins such as p21^Waf1/Cip1^, cdc2, and cdc25C	[86]
		ISS/ RWV	Human PBMC	T cells had significantly downregulated gene expression of Rel/NF-B transcription factors	[87]
		2D/ parabolic	Human PBMC	Anti-CD28/conA activated T cells had decreased Zap-70 expression	[89]
		Sounding Rocket/Parabolic Flight/2D Clinostat	Jurkat T cells	5 genes remained unaltered in all microgravity conditions	[94]
		Sounding Rocket/2D Clinostat	Jurkat T cells	11 transcript clusters in non-activated Jurkat T cells were altered	[95]
		Spaceflight/RWV and RPM	Mouse Primary	T cells had suppressed immune regulatory genes compared to controls	[96]
B cells	Simulated	RWV	HMy2.CIR	S decreased ion-radiation induced cell survival and increased apoptosis	[105]
	Real	Parabolic Flight	Human PBMC	A decrease in B cells	[44]
		Spaceflight	Human PBMC	15-day spaceflight showed no changes in B cell levels	[46]
		Spaceflight	Mouse Primary	A significant reduction in B cells in the spleens of mice	[104]

## 4. Future Directions and Discussion

### 4.1. Is There a Link between Microgravity and Mechanotransduction?

**Figure 3:**
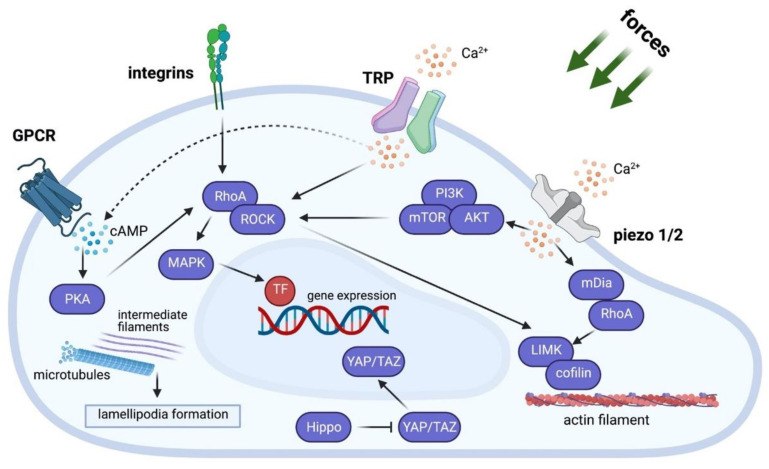
A simplified overview of the mechanotransduction pathways. Illustration depicting the major mechanotransduction pathways that are influenced by force and ultimately affect cell gene expression.

From these reviewed studies, it is apparent that microgravity influences immune cells in a variety of ways. Many of the effects of microgravity could be due to the lack of gravitational force on the cells. In order to understand how microgravity is able to affect cells, it is important to know how cells sense forces acting on them. A process called mechanotransduction allows cells to convert mechanical forces (e.g., from the surrounding extracellular matrix or biomaterials) into biochemical signals that then induce downstream pathways [23]. Briefly, detection of these forces occurs through G-protein coupled receptors (GPCRs), integrins, and mechanosensitive ion channels that then transduce the force into biochemical or electrical signals within cells [106] (Figure 3). The transient receptor potential (TRP) ion channel superfamily and the Piezo ion channel family are two of the most important ion channels in mechanotransduction [107,108]. These receptors and channels initiate a variety of signals within the cell affecting molecular pathways and ultimately, gene expression. Comprehensive reviews on mechanotransduction can be found in these cited papers [107,108,109,110,111,112,113,114,115] (Figure 3). It is reported that gravitational force plays an important role in regulating cell processes and any reduction in this force (for example in the form of microgravity) shifts the balance and homeostasis within cells [22]. Future work should focus more on how mechanotransduction pathways are affected under microgravity conditions. This can be achieved using RNA-Seq and cytoskeleton inhibitors, e.g., cytochalasin D, blebbistatin, and ML-7, to probe whether and which mechanotransduction pathways are involved in cellular sensing of microgravity [116,117,118].

### 4.2. Technological Advances for Future Microgravity Research

**Figure 4:**
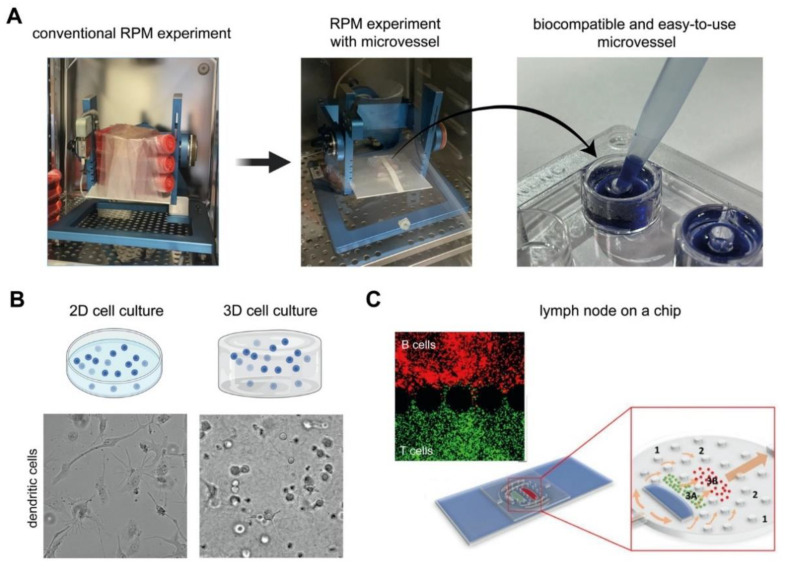
Cell culture techniques for improved physiological relevance in microgravity research. (**A**) Biocompatible and easy to use microvessels developed for high throughput studies [119]. (**B**) 2D and 3D biomimetic cell culture model showing more rounded cells in 3D culture compared to 2D [69]. (**C**) Organ on a chip technology by combining 3D cell culture and microfluidic systems [120].

The extent by which immune cells have been studied in microgravity revealed differences, at times contradicting, immune signatures at the genetic, protein expression, cytokine secretion, and functional level. This is still the tip of the iceberg and similar levels of research have to be furthered for all the other immune cell types reviewed here, and similarly at the multiculture and organoid levels, to fully comprehend the immune system in microgravity conditions. Although immunological research in microgravity appears to be straightforward, there are some limitations of the current techniques and handling to be addressed. Especially, current methods for using simulated microgravity platforms can limit the scope of investigation if large cell culture vessel sizes are used (e.g., T25 and T75 flasks). The large vessels (e.g., cell culture flask) sometimes used in simulated microgravity conditions require high cell numbers and copious volumes of media. Furthermore, the rotation speed of simulated microgravity platforms largely changes between platforms, ranging from a few degrees per second to tens of revolutions per minute (i.e., >3600 deg/s), which might cause slight discrepancies between platforms. As discussed above, many experiments in microgravity research were performed using cell lines rather than primary cells. A need for smaller cell culture vessels is therefore required, especially for the study of non-proliferating primary immune cells. Recently, biocompatible and easy to use microvessels have been developed for use in simulated microgravity allowing for more high throughput studies to be performed (Figure 4A) [119]. Although the advantage of using cell lines avoids genetic variability between donors, they cannot fully represent the biological significance of primary cells (e.g., macrophages from PBMC vs. THP-1). Besides that, contradictory results could be observed for both primary cell and cell lines due to differences in cell differentiation protocols. A standardized differentiation protocol could make the obtained data comparable between research groups. In addition, most of the experiments have addressed the alteration in cellular response in a short period (within several hours). Long term studies (days or weeks) will increase the significance impact and enhance translational possibilities.

Growing evidence points towards a key role of the extracellular matrix in the modulation of the potency of immune cells [52,69,121,122,123]. Traditional 2D cell culture plastics (e.g., microwell plate, Petri dish, and cell culture flask) are unable to resemble the in vivo situation [124,125]. The main advantages of 2D tissue plastic cell culture are easier environmental control cell observation, measurement, and eventual manipulation in comparison with 3D cell culture models. As a major consequence non-physiological cell behavior and failure in the translation of cell culture results have been reported [126,127,128,129,130]. To better simulate the in vivo situation, more complex 3D models have been introduced for example using collagen, alginate, or PEG [38,131,132,133,134]. The 3D models should span the gap between traditional 2D cell culture plastic and an animal model by mimicking key features of the native microenvironment. The additional dimensionality of 3D culture leads to the differences in cellular responses because of the spatial organization of the cell surface receptors and the physical constraints to cells. As an example, the morphology of dendritic cells is different in 2D culture and 3D culture in collagen matrices [69] (Figure 4B). Although few reports utilized 3D cell culture models to study cells under microgravity [135,136,137], no work has been done with regard to immune cells in 3D under real microgravity or simulated microgravity. The 3D scaffolds could benefit the study of immune modulation in wound healing and tissue regeneration [138,139], which could be drastically different during spaceflight. Besides 3D cell culture models, microfluidic devices could enhance the understanding of the immune system in microgravity as immune cells are always exposed to shear flow, during inflammation within interstitial tissues or within lymph nodes. As an example, an engineered lymph node on a chip could enable the study of the adaptive immune response during infection or allergy, as well as can be used as a testing platform for drug screening [120,140,141] (Figure 4C). Combining these novel technologies with microgravity platforms could be used to uncover immunotherapy drug targets and provide further insight into disease specific immune responses. Research has shown that microgravity has a positive impact on combating diseases such as cancer by regulating cancer cell proliferating and survival [142,143,144]. The mechanisms found can be used to benefit cancer treatment on Earth. Microgravity is increasingly being used as a unique platform for assisting in drug discovery and development [145], as well as for implementing personalized medicine treatments [146].

Many key discoveries in cell biology would not have been possible without microscopy. Currently, bright-field microscopy can provide multi-scale spatio-temporal studies of cell migration [147], proliferative behavior [148], and cell mechanics [149]. In particular, single cell mechanics is of interest in modern cell biology and immunology. It allows prediction of pathogen infected cells [150], maturation stage of dendritic cells [151,152], and T cell and antigen-presenting cell interactions [153,154]. Cell mechanics has been hypothesized to alter as an adaptation mechanism to microgravity since it might be correlated to cytoskeleton remodeling [55,56,58]. Combining the 3D cell culture models with a live imaging platform in microgravity conditions will allow observation of cells in their native stage with minimal cell manipulation at the single cell level. While microscopy systems are available onboard the ISS, in parabolic flights or suborbital rockets [155], simulated microgravity platforms generally lack this feature. So far, only clinostats rotating around one axis or a specialized custom-made RPM incorporating a digital holographic microscope have cell imaging capabilities [156]. Recently, a microscope has been implemented for use on simulated microgravity platforms [157]. However, there is still room for improvement in terms of automation, resolution of images, and having depth capabilities in imaging 3D culture models.

Overall, the study of immune cells under microgravity conditions has been studied for tens of years. Despite the knowledge that we already have, there is a long way to go before we fully comprehend the dynamic network of the immune system under microgravity. More studies should be done to understand the interactions between cells of the immune system in microgravity and how they are affected as a whole system in vivo. Most importantly, standardized protocols are needed for studying the effects of both real and simulated microgravity.

## Data Availability

Not applicable.
